# Premenstrual syndrome and factors associated with it among secondary and preparatory school students in Debremarkos town, North-west Ethiopia, 2016

**DOI:** 10.1186/s13104-019-4549-9

**Published:** 2019-08-22

**Authors:** Abebaw Abeje, Zerihun Berhanu

**Affiliations:** 1 Department of Midwifery, College of Medicine and Health Sciences, Hawassa University, PO Box- 1560, Hawassa, Ethiopia; 2 Department of Reproductive Health, School of Public Health, College of Health Sciences and Medicine, Dilla University, Dilla, Ethiopia

**Keywords:** Premenstrual syndrome, Magnitude, Factors, Students

## Abstract

**Objective:**

The purpose of this study was to assess the magnitude of premenstrual syndrome and its associated factors among secondary school students in Debremarkos, North west Ethiopia.

**Results:**

Premenstrual syndrome was reported by 81.3% of the participants. Statistically significant association was observed between the occurrence of PMS and age, AOR (CI) 1.20 (1.01, 1.44), involving in heavy non-academic duties, AOR 95% (CI)  2.13 (1.17, 3.89), early menarche (< 12 years), AOR (95% CI) 2.68 (1.32, 5.47) and long menstrual cycles (> 35 days), AOR (95% CI) 0.13 (0.02, 0.75).

## Introduction

Premenstrual Syndrome (PMS) is a collection of physical, cognitive, affective and behavioral cyclically occurring symptoms during the luteal phase of the menstrual cycle and resolve at or within a few days after the onset of menstrual flow [1]. Though more than 200 symptoms have been known to occur, the most frequently occurring symptoms include headache, fatigue, bloating, backache, breast tenderness, food cravings, fatigue, anxiety, irritability, social withdrawal and depression [1–5]. Premenstrual syndrome for most women started at their age of menarche [6].

More than 90% of females all over the world experience these symptoms during their child bearing age [1]. However, a more severe form of Premenstrual Syndrome (PMS), premenstrual dysphoric disorder (PMDD), which leads a significant loss of function due to unusually severe symptoms occurs in 2–6% of women [7]. According to a cross sectional study in Jimma University, 99.9% students had at least one premenstrual symptom in many of their cycles [8].

Various socio-biological and psychological factors such as hormonal change, diet and lifestyle have been proposed to cause PMS. PMS affects the daily life of menstruating women of any age; race; and part of world [1, 9].

Premenstrual Syndrome results a significant psychosocial dysfunctions as the symptoms often coexist with anxiety and other psychological symptoms [10, 11]. It is the important cause for adolescent girls to frequently miss classes and exams, to score a lower grade and even withdrawal from their learning [6, 8, 12]. It further interferes with the development of nations by increasing mood instability and decreasing daily activity among women [6, 13].

In spite of these facts about premenstrual syndrome, little attention have been given specifically in developing countries like Ethiopia. Therefore, the aim of this study was to assess the magnitude of premenstrual syndrome and identify its predisposing factors among secondary and preparatory school students in Debremarkos town.

## Main text

### Methods

A facility based cross sectional study was employed in Debremarkos town which is located 300 km Northwest of Addis Ababa, the capital of Ethiopia from Oct 11 to Oct 26, 2016. According to the 2007 Ethiopian population and housing census, there was an estimated total population of 62,469 in the town of which 52.1% were females. Fourteen thousand six hundred eighteen were reproductive age women [14]. In the town, there are 04 governmental and 01 private secondary and preparatory schools. The total number of students is 7483 of which 3564 are females.

Single population proportion formula was used to calculate the sample size under the assumptions of 75.4% proportion of premenstrual syndrome in Dabat and Kolla diba [15], 95% confidence interval (CI) and 4% precision. After adding 10% possible none response rate, a total of 496 students were enrolled.

Grades 9 to 12 female students from all the schools in the town were included in the study. Sampling frame was constructed by taking the list of all female students with their respective grades and sections from each school. Simple random sampling technique was used to select the study subjects.

Participants who have at least one of the somatic and affective symptoms occurring from 13 days before to 4 days after the onset of menses were considered to have PMS [16]. The participants’ habit of physical activity was measured by their personal report of not at all, irregularly or regularly. Daily allowance was considered adequate or inadequate based on their personal report. Sugar intake was classified as excessive when a participant’s daily table sugar intake is greater than 12 teaspoons, moderate when it is 6–12 teaspoons; and restricted/minimal when it is less than 6 teaspoons [17].

Non-academic duty: students no duty at home or outside were grouped in “not at all”; those involving in activities such as cleaning the house, making coffee, bed making, washing own clothes, were classified in “simple” category; on the other hand, students participated in daily labor, marketing, cooking and farming activities were categorized in “heavy” group.

Menstrual cycles were heavy when a lady changes 5 or more sanitary pads a day and scant if one or less.

Self-administered questionnaire containing socio-demographic information, life style and behavioral issues and reproductive and menstrual pattern issues was used for data collection.

The tool was pre-tested on 5% of the sample size, (25 students) in Dembecha high school and important amendments were made accordingly. Data collectors and supervisors were trained on the overall steps and procedures of data collection and proper data handling techniques.

Four diploma and one degree female teachers were recruited for data collection and supervisor respectively. Informed consent was taken from each participant just before data collection. Data were and coded prior to data. A templet was prepared in Epi-data version 3.1 for data entry and SPSS version 22 was used for data analysis.

Simple binary logistic regression analysis was employed. First, was computed for each predictor variable. All independent variables with P value < 0.2 in the bivariate analysis were included in the multivariate analysis. P value of less than < 0.05 at 95% CI was considered as statistically significant.

### Results

Totally, 496 female students were participated in this study making a response rate of 99.2%. Four incomplete questionnaires were omitted from the analysis.

#### Sociodemographic characteristics of the respondents

Four hundred twenty-seven (86.8%) of the study participants were urban dwellers. The study participants mean age was 17.61 ± 1.93 years old ranging from 14 to 24 years. Most of them, 442 (91.3%) were 15–19 years adolescents. Majority of them, 489 (98.6%) and 471 (95.7%) of them were Amhara in ethnicity and Orthodox Christianity by religion, respectively. The family size where participants living with ranges from 1 to 14 with the mean of 5.31 ± 2.26. Two hundred sixty-three (53.5%) of the study participants didn’t involve in any physical activity. Sixty-four (13.0%) of them have been involved in heavy non-academic duties in the family. None of the respondents had smoked and used chat (Table 1).

**Table 1:** Socio demographic characteristics of secondary and preparatory school female students in Debremarkos town, North West Ethiopia, 2016

Variables	Frequency (n = 492)	Percent **(%)**
Age; n = 484		
10–14	4	0.8
15–19	442	91.3
20–24	38	7.9
Marital status		
Single	478	96.3
Ever married	18	3.7
Family history of PMS^a^		
Yes	193	39.2
No	229	46.5
I don’t know	70	14.2
Physical activity		
Not at all	263	53.5
Irregularly	170	34.6
Regularly	59	12.0
Nonacademic duty in the family; n = 491		
Not at all	87	17.7
Simple	340	69.1
Heavy	64	13.0
Coffee intake (No of cups/day)		
0	239	48.6
1–4	185	37.6
≥ 5	68	13.8
Tea intake		
Yes	471	95.7
No	21	4.3
Drinking alcohol		
Not at all	438	89.0
Irregularly with occasions	35	7.1
Regularly	19	3.9

^a^PMS: Premenstrual syndrome

#### Reproductive characteristics and menstrual patterns of the participants

The participants’ age at menarche ranges from 10 to 17 years with a mean of 13.23 ± 1.47 years. More than one-third (34.9%) of the respondents experienced early menarche, before celebrating their 12th birthday. Though only 1 (2.6%) had pregnancy history, 38 (7.7%) of respondents were sexually active prior to data collection. The mean cycle length was 28.44 ± 2.45 days. The duration of menstrual flow was from 1 to 9 days with the mean of 4.33 ± 1.51 days (Table 2).

**Table 2:** Reproductive characteristics and menstrual patterns of secondary and preparatory school female students in Debremarkos town, North West Ethiopia, 2016

Variables	Frequency (n = 492)	Percent (%)
Age at menarche		
9–12	171	34.9
13–14	184	37.6
15–17	135	27.6
Menstrual cycle pattern		
Regular	371	75.4
Irregular	121	24.6
Menstrual cycle length in days (n = 371)		
< 21	10	26.6
21–35	353	71.7
> 35	8	1.6
Duration menstrual of flow in days		
1–2	18	3.7
3–6	398	80.9
≥ 7	76	15.4
Amount of menstrual flow (n = 477)		
Heavy	168	35.2
Normal	275	57.7
Light	34	7.1

#### Patterns of premenstrual symptoms

According to the ACOG definition, 402 (81.3%) of the participants had premenstrual syndrome during their preceding two consecutive cycles. Anger (68.5%) and abdominal bloating (73.4%) were the most frequently observed affective and somatic symptoms respectively. Other affective symptoms including anxiety (24.0%), social withdrawal (44.9%), confusion (34.1%), depression (43.3%) and irritability (16.1%) as well as somatic symptoms such as headache (23.0%), tender breasts (4.7%) and swelling of extremities (25.0%) were also among the observed premenstrual symptoms.

#### Factors associated with premenstrual syndrome

In this study, the existence of PMS had a statistically significant association with age, AOR (CI) 1.20 (1.01, 1.44), participating in heavy non-academic duties, AOR 95% (CI) 2.13 (1.17, 3.89), coffee intake, AOR (CI) 1.93 (1.12, 3.31), early menarche, AOR (95% CI) 2.68 (1.32, 5.47), long menstrual cycles (> 35 days), AOR (95% CI) 0.13 (0.02, 0.75), and being sexually active, AOR (95% CI) 5.03 (2.20, 11.51) (Table 3).

**Table 3:** Bivariate and multivariable analysis of factors associated with premenstrual syndrome among secondary and preparatory school students in Debremarkos town, North West Ethiopia in 2016

Variables	PMS		Crude OR (95% CI)	AOR (95% CI)
	No	Yes		
Age (years)	90	402	1.27 (1.09, 1.48)	1.20 (1.01, 1.44)*
Nonacademic duty				
Not at all	26	61	1.00	1.00
Simple	7	57	3.47 (1.40, 8.62)	2.46 (0.92, 6.60)
Heavy	57	283	2.12 (1.23, 3.63)	2.13 (1.17, 3.89)*
Physical activity				
Not at all	39	224	2.73 (1.43, 5.19)	1.01 (0.57, 1.77)
Irregularly	32	138	2.05 (1.05, 3.40)	0.49 (0.25, 1.02)
Regularly	19	40	1.00	1.00
Coffee intake in cups				
0	59	180	1.00	1.00
1–4	29	156	1.76 (1.08, 2.89)	1.93 (1.12, 3.31)*
≥ 5	2	66	10.8 (2.57, 45.5)	6.79 (1.50, 30.77)*
Age at menarche (years)				
9–12	16	155	3.14 (1.69, 5.79)	2.68 (1.32, 5.47)**
13–14	45	139	1.00	1.00
15–17	28	107	1.24 (0.73, 2.11)	1.09 (0.60, 1.99)
Menstrual pattern				
Regular	72	299	1.00	1.00
Irregular	18	103	1.38 (0.79, 2.42)	0.49 (0.07, 3.29)
Menstrual cycle length				
< 21	19	112	1.38 (0.79, 2.40)	2.26 (0.35, 14.57)
21–35	67	286	1.00	1.00
> 35	4	4	0.23 (0.06, 0.96)	0.13 (0.02, 0.75)*
History of intercourse				
Yes	14	24	1.00	1.00
No	76	378	2.90 (1.44, 5.86)	5.03 (2.20, 11.51)**

*≥ P < 0.05; **≥ P < 0.001

### Discussion

The prevalence of premenstrual syndrome was 81.7% with 95% confidence interval of (78.3–85.0%). This finding is less than that of reported in cross-sectional studies conducted in Addis ketema preparatory school [6] and Jimma University [8], Ethiopia, which reported 86.1% and 98.2% respectively. It is also less than 99.6% reported among Iranian female university students [18]. These differences might be due to small sample sizes used for the first two studies and sociocultural variations as Debremarkos is an area of a culture in which people especially young females did not report problems genuinely. This observed difference can also be explained by the fact that the severity and incidence of premenstrual symptoms increases with age till 20 to 24 years. The mean age of the participants in this study is lower than that of the mentioned studies.

On the other hand, this finding was higher than findings like 55.8% in Egypt [9], 61.5% among students in a rural school of West Bengal, India [4], 64.6% among Japanese high school students [10] and 36.3% in Sistan and Baluchestan University, Iran [19]. This is probably because of population difference, high school versus university students or sociocultural differences in thresholds.

However, similar findings were reported in Mekelle University, Ethiopia [20] and Medical Students in Al Qassim University, KSA [19] which reported 83.2% and 78.5% prevalence of PMS respectively.

In this study, statistically significant association was observed between age of the participants and the occurrence of premenstrual syndrome. Statistically significant association was also observed between the occurrence of premenstrual syndrome and academic year among Addis ketema preparatory school students [6]. However, a lower prevalence of premenstrual syndrome was reported among Japanese high school students than that in adult women [10]. This may be the socio-demographic differences between the study populations.

Participating in heavy non-academic duties in the family was found to have statistically significant association with the occurrence of PMS. This finding is supported by a report showing a statistically significant association with mother’s occupation in Bengal, India [4]. This may also be related with a statistically significant association between physical activity and PMS observed in West Bengal [21]. These all may because participating in such heavy duties may add extra stress on the students which may compound the depressive nature of the premenstrual period.

The occurrence of PMs in this study had statistically significant association with long menstrual cycles (> 35 days). This is supported by a study in West Bengal, India which identified menstrual cycle intervals and amount of blood flow during menstruation as important predictors of PMS [4]. Furthermore, early menarche (< 12 years) in this study was found to be statistically significant with PMS. On the contrary, duration of menstrual flow was not statistically significant in relation to severity of PMS in Egypt [12]. This may be due to the fact that psychological effect of menstruation. This is probably because of a difference in study populations in which the relationship is influenced by other sociodemographic and sociocultural variables.

## Limitations of the study

In this study, most variables including the outcome variable were measured by the participants’ subjective-report. This may lead to the introduction of observation and recall bias. When variables are measured by the participants’ verbal report, the measurements may not be consistent for all of the participants, problems for certain individuals may not be a problem for the others or some may not recall the actual events occurred. The other most important thing for the readers to consider is the causal relationship was not confirmed in this study, we would rather recommend further studies using better designs.

## Data Availability

We do not want to share the raw data as we have an ongoing related research project.

## Abbreviations

AOR: adjusted odds ratio
CI: confidence interval
OR: odds ratio
